# Birth Order and First Sexual Experience: Do Siblings Influence Sexual Debut in Adolescents?

**DOI:** 10.1007/s10508-021-01979-w

**Published:** 2021-08-21

**Authors:** Marta Pasqualini, Amanda Sacker, Anne McMunn

**Affiliations:** 1 Observatoire Sociologique du Changement, Sciences Po, 75007 Paris, France; 2 Department of Epidemiology and Public Health, University College of London, London, UK

**Keywords:** Adolescent sexual behaviors, Birth order, Child siblings, Family relations, Structural equation model

## Abstract

Birth order may foster specific roles for individuals within the family and set in train a dynamic that influences the development of specific behaviors. In this paper, we explored the relationship between birth order, sex, timing of sexual initiation, and its consequences for risky sexual behavior and sexual health. We conducted a path analysis to simultaneously estimate direct and indirect effects using data from the National Survey of Sexual Attitudes and Lifestyles (NATSAL-3). Whereas women born as only-children were more likely to sexually debut at later ages, middle-child boys were significantly more prone to initiate sexual intercourse earlier compared with first-borns. As expected, early sexual initiation was associated with riskier behaviors and sexual health outcomes. These associations were partially moderated by siblings role as confidants about sexuality. Our findings have implications for preventive programs aimed at promoting healthy sexual debuts and behaviors over the life span.

## Introduction

### Early Age at First Intercourse, Sexual Behaviors, and Sexual Health

In post-industrial societies, unplanned pregnancy, abortion and sexually transmitted infections (STIs) are of major public health concern (Johnson et al., 2005). Among developed countries, England has the highest rate of unintended pregnancy after the U.S., with 45% of pregnancies being unplanned at the time of conception (Gov. UK, 2018a, b) and an even higher age-standardized abortion rate equal to 17.4 per 1,000 women (Gov. UK, 2018a, b) compared to that registered in the U.S. with a 13.5 ‰ rate (Guttmacher Institute, 2019). Among other factors, early age at first intercourse has been considered as a powerful determinant of poor sexual health (Bradley-Stevenson & Mumford, 2007), and in the UK, nearly a third of men and a 26 per cent of women aged 16–19 first had sexual intercourse before the age of 16 (Guttmacher Institute, 2019). Although there is a lack of a consistent and comprehensive conceptual framework that systematizes our understanding of adolescent and young adult sexual behaviors, it is possible to draw from the literature two sets of risks associated with early sexual experience. Evidence from previous work has suggested that youths who have sex early in life take more physical sexual risks. For example, early initiators are less likely to obtain, negotiate, and use contraception and are likely to have more life-time sexual partners than later initiators (e.g., Finer & Philbin, 2013; Sneed, 2009). This behavior leads them to have a greater risk of contracting STIs as well as unplanned pregnancies (e.g., Finer & Philbin, 2013; Sneed, 2009). Another set of risks associated with early initiation of sexual activity has been mainly related to the psycho-social context. Indeed, early initiators are more likely to have had non-consensual sex, due to a lack of control in the relationship, and to an internalized pressure to become sexually active (Finer & Philbin, 2013). Early initiators are also at greater risk of having experienced sexual violence (Finer & Philbin, 2013). Individual characteristics and personality traits, such as being impulsive and sensation seeking, have been shown to be associated with early sexual initiation and later sexual risk behaviors (e.g., Spitalnick et al., 2007). Moreover, adolescent uninhibited behavior seems to have long-term effects on problem behaviors, including sexual risk taking (Epstein et al., 2014).

While the family is likely to be the early developmental context in which norms and behaviors related to sexual activity are first learned, research has almost exclusively focused on parents (Grossman et al., 2016; Overbeek et al., 2018), potentially underestimating the important role of siblings as a source of intra-family influence (Elton et al., 2019; Killoren et al., 2019; Killoren & Roach, 2014).

### The Sibling Effect

Birth order represents a social determinant of individual development, which strongly affects the propensity to adopt certain behaviors and attitudes. Indeed, given competition between siblings for parental attention and resources, individual siblings try to distinguish themselves from one another in order to establish a unique familial niche from which to elicit parental attention (Hertwig et al., 2002; Sulloway, 1996). Indeed, differences in parental investment and competition for power, attention and personal gain is a result of the natural hierarchy developed when a new-born comes into the family (Cole, 2014; Sulloway, 2007). Siblings receiving more parental investment are expected to have more positive emotions toward the family and less unconventional behaviors compared with siblings receiving less parental attention (Bu & Sulloway, 2016; Hertwig et al., 2002). Thus, with the aim of increasing parental attention, younger siblings may develop attitudes and qualities that generally make them more rebellious, more open to new experiences and more likely to adopt risky behaviors. This type of differentiation from the oldest sibling enables later-born children to solicit a different type of attention and investment from parents and, thus, avoid direct rivalry with other siblings.

Sulloway (1996, 2007) developed the hypothesis concerning the relationship between birth order and behavioral dispositions by arguing that it is not the rivalry itself, but siblings’ strategies to reduce this competition that leads them to pursue differing ways of optimizing parental investments (Sulloway, 2007). Strategies adopted with the aim of safeguarding parental attention often diverge according to birth order. As first-borns begin life as only children and thus are not born into experiences of sibling rivalry, they display beliefs and personality traits that mirror their parents and are generally more likely to be responsible and conform to parental authority, while later-borns often behave in the opposite way (Sulloway, 1996, 2007). Sulloway’s approach has been called “niche portioning” since it suggests that while first-borns act as surrogate parents, later-borns are family newcomers seeking an open niche within the family (Hertwig et al., 2002). Empirical controlled studies have reported that first-borns are more conscientious and more responsible than later-borns who, by contrast, appear to be more agreeable, accommodating and affectionate (Healey & Ellis, 2007). However, a relevant difference between middle children and the last-born in families with more than two children has been suggested, in that first- and last-borns will both see their parents and familial resources as dependable sources of support to a greater degree than will middle-borns (Bu & Sulloway, 2016; Salmon & Daly, 2002). Moreover, a large body of literature investigating consequences of birth order on individual behaviors and attitudes has revealed consistent birth order differences for many traits and behaviors such as antisocial behaviors (e.g., Bank et al., 2004) and reproductive choices (e.g., Milne & Judge, 2009). Despite some criticism of Sulloway’s approach, successful replication lends support for the credibility of his theory (Eckstein et al., 2010; Healey & Ellis, 2007), arguing that sibling influences on individual characteristics are largely due to structural factors such as rivalry, differential treatment and resource allocation.

However, empirical evidence has also suggested that siblings are major socializing agents with regard to issues that are relevant for adolescents, such as the first sexual experience. Siblings are likely to share similar sexual experiences, such as age at first intercourse, the degree of intimacy and attitudes about sex (Haurin & Mott, 1990; McHale et al., 2012; Widmer, 1997), and this may be also explained by social learning theory (Bandura, 1992). Indeed, siblings observe, imitate and model their behavior on each other. By colluding and aligning to resist the vertical influence of parents, they use each other for social referencing (Bandura, 1992). Therefore, the presence or absence of contact and interaction with siblings may be fundamental in shaping individual beliefs and behaviors. We have seen that the mechanism of differentiation between siblings can influence individual personality characteristics and attitudes, possibly increasing the probability of adopting risky behaviors for later-borns. Indeed, since horizontal ties—such as those between siblings—are characterized by greater perception of similarity compared with that between parents and children, having siblings represents a direct influence on individual development, as they may serve as social partners and role models (Bandura, 1992; McHale et al., 2012).

Overall, research suggests that the level of intimacy and the relational balance of power within sibling dyads is dependent on their sex composition (Furman & Lanthier, 2002). In general, same-sex siblings are more likely to report more access to shared life events than opposite-sex siblings, which might increase younger siblings’ early sexual engagement (Elton et al., 2019). However, some research has argued that what has been considered as a “same-sex effect” is better described as a “sister-effect” (Widmer, 1997) since sisters’ dyads may be particularly important in the socialization of adolescent sexuality due to their greater levels of intimacy, on average (Killoren et al., 2019; Widmer, 1997).

Finally, older siblings may also serve as mentors since they can provide information concerning safe sexual activities and set standards of conduct more effectively than parents (Elton et al., 2019; Killoren & Roach, 2014; Killoren et al., 2019). This suggests that adolescents who discuss sexual matters with siblings enter into sexual activity with greater confidence and a deeper level of knowledge concerning sexual risks, which may help them to avoid risky behavior regardless of their age of initiation.

Siblings are not only a direct and passive source of influence and learning, but they may also serve to transmit, validate and reinforce attitudes, norms and beliefs through communication which plays as a mechanism of sexual socialization (Christoper, 2001). According to symbolic interactionism (Christoper, 2001), siblings’ interactions and conversations about sexuality are a mechanism that shapes sexual roles and ways of learning about behaviors (Killoren et al., 2019). According to this theoretical approach, explicit communication about sex is a way to derive-meaning, add value and shape attitudes to one’s own sexual experiences as well as a way to transfer them to another person (Christoper, 2001; Killoren et al., 2019).

The aim of this paper is to investigate the relationship between birth order, sex, risky behavior and sexual health, and the role of siblings in these associations by addressing the following research hypothesis:

#### Hypothesis 1

Given the evidence of the effect of birth order on behavioral characteristics (Adler, 1937; Sulloway, 2007), later-borns will experience their first intercourse earlier than first-borns (with middle-born siblings being the earliest), and age at first intercourse will predict risky sexual behavior that, in turn, contributes to later sexual health issues (**Hypothesis 2**).

#### Hypothesis 3

The evidence reviewed suggests that dyads of same-sex siblings are more likely to create coalitions and report more access to shared social life events that likely increase opportunities for the earlier sexual debut of younger siblings (Killoren & Roach, 2014). Thus, we hypothesize that the sex composition of siblingships will moderate birth order effects on first sexual initiation (Widmer, 1997), with later-borns of same-sex siblings being more likely to have an earlier sexual debut than later-borns of opposite-sex siblings (“same-sex siblings effect”) and that this will especially occur if both siblings are female (“sister effect”).

#### Hypothesis 4

As prior studies have argued that sisters are more likely than brothers to be influential (Tucker et al., 1997) and to serve as confidants and as mentors about sexuality (Killoren & Roach, 2014; Killoren et al., 2019), we hypothesize that respondents reporting having used sister(s) as referent persons for learning about sexual activity will predict safer sexual habits by moderating the association between early age at first intercourse, risky behaviors and sexual health. Indeed, we expect to find a lower probability of having adopted risky behaviors among both men and women who had a sister as a confidant about sexuality, regardless of their age at first intercourse (“mentor effect”).

## Method

### Participants

The data used in this paper are drawn from the third and most recent wave of the National Survey of Sexual Attitudes and Lifestyles (NATSAL-3), a cross-sectional dataset providing detailed information on the sexual behavior of more than 15,000 adults aged 16–74 randomly selected in the UK between September 2010 and August 2012. Young people aged 16–34 were over-sampled in order to provide sufficient statistical power to examine behaviors among the age-group at highest risk for a range of sexual health outcomes (Erens et al., 2014). The response rate in terms of completed interviews is about 57.7% for Natsal-3 (Erens et al., 2014).

**Fig. 1:**
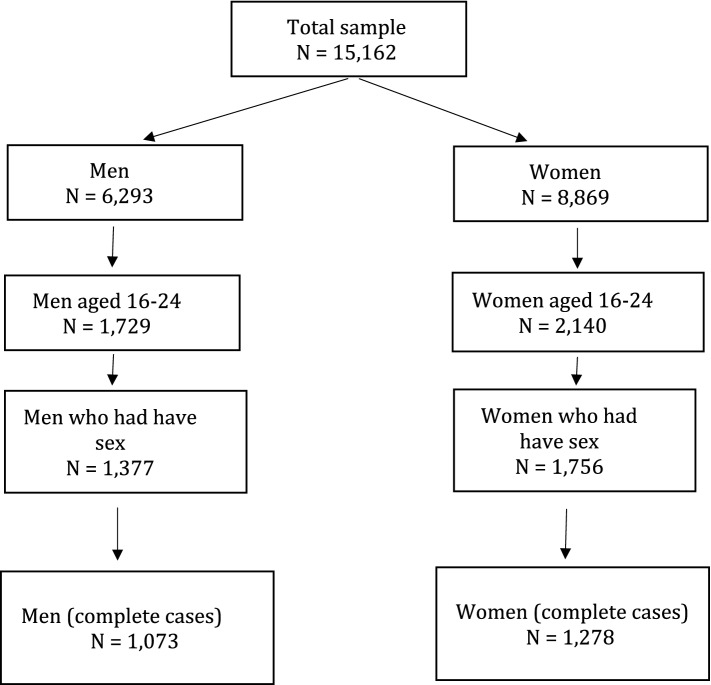
The sample selection

We used survey functions in order to account for the sampling design of the Natsal-3 dataset. Figure 1 provides a flowchart of the selection of the analytic sample which was restricted to young adult respondents aged 16–24 who were already sexually active at the time of interview (more than 80% of the young adults) and for whom we do not have any missing values. Since outcomes of interest refer to events that mostly happened during adolescence, our age-related restriction should reduce recall bias. After these restrictions, we have a total analytic sample composed of 1,073 males and 1,278 females. Natsal-3 was granted ethical approval from the Oxford A NHS Research Ethics Committee (reference: 09/H0604/27).

### Measures

Information was collected in face-to-face interviews and in individual self-completion questionnaires.

#### Birth Order

Participants were asked to think about the sibling(s) they lived with when they were growing up, and whether they were the oldest, the youngest, in between or an only child.

#### Age at First Intercourse

To look at the timing of sexual initiation, we focus on the first heterosexual vaginal intercourse. The survey asked respondents to indicate at what age they first had sex resulting in a continuous variable (range 13–24).

#### Risky Behavioral Outcomes

We consider three aspects of risky sexual behavior. Whether the first sexual experience was consensual or not is included as a measure of psycho-social risk. Specifically, participants were asked whether they were totally willing to have intercourse the first time or whether they felt persuaded or even forced to have sex, this was considered as a proxy of consensual sex. Not using any contraceptive method at the first intercourse is considered risky in terms of being vulnerable to unplanned pregnancy as well as to STIs and diseases. Respondents were asked whether they had used any reliable form of contraception at that time (specifically: condoms, pill, emergency contraceptives or other). Participants were asked the number of sexual partners they had in the last year. Number of partners was dichotomized as two or more based on the definition provided by the Centers for Disease Control and Prevention, suggesting multiple sexual partners are an indicator of risky sexual behavior in adolescents and young adults.

#### Sexual Health Outcomes

Participants were asked whether they were ever diagnosed with a STI including genital warts, genital herpes, gonorrhoea, chlamydia, trichomonas, syphilis, nonspecific or non-gonococcal urethritis or whether they were ever diagnosed with a STI but cannot remember which one. Female respondents were also asked whether they had any pregnancies in the last year, whether it/they was/were planned or not, and whether they had ever had a pregnancy that ended with a voluntary termination.

#### Sex Composition of Siblingships

Dummy coded variables have been constructed to account for the composition of respondents’ siblingship. Specifically, they identify respondents having one or more brother(s) but no sister(s) (equal to 1 and 0 otherwise); those having one or more sister(s) but no brother(s); and those having mixed-sex siblings. According to respondents’ sex, we renamed them as “only opposite-sex sibling(s)”; “only same-sex sibling(s)”; “mixed-sex sibling(s).”

#### Siblings as Source of Information About Sex

Respondents were asked to indicate who was their source of information about sex (doctor, nurse or clinic; internet; lesson at school; books/magazines/newspapers; friends; television/DVD/videos; mother; father; sexual partner; pornographic materials; brothers/sisters). We generated a dummy variable taking value 1 when they reported sibling(s) as their reference person(s) in sexual matters and 0 otherwise (reference category). Then, in order to test any “mentor effect” we further disaggregated those who reported sibling(s) as their source of information about sex into those who had only same-sex sibling(s) = 1; those who had only opposite-sex sibling(s) = 2; and those who had mixed-sex sibling(s) = 3. Respondents having no sibling(s) (7% of the sample) and those who had siblings but who did not report them as source of information about sex were used as the reference category.

#### Covariates

A set of demographic control variables were also used in the model such as the respondents’ age at the interview in years, the family size (3 or more siblings vs 2 or less), religion (None; Christian—Church of England/Anglican; Christian—Roman Catholic; Christian—other; Non-Christian) and ethnicity (White/White British; Mixed ethnicity; Asian/Asian British; Black/Black British; Chinese /Other). Moreover, we used household income in £ (< 2,500; 2,500–4,999; 5,000–9,999; 10,000–19,999; 20,000–29,999; 30,000–39,999; 40,000–49,999; 50,000 +) and whether the participant lived more or less continuously with both parents at home until age 14 as proxies of the socio-economic background. Other covariates relating to the first sexual experience included in the model were: having smoked cannabis (Yes/No) before having sex the first time; been a bit drunk before having sex the first time (Yes/No); and if in a stable relationship at first intercourse (Yes/No). These variables were useful in controlling for specific circumstances in which first sexual experience occurred as they have been widely recognized as significant risk factors for unwanted and unprotected sex (Guleria et al., 2019; Scott-Sheldon et al., 2016). Indeed, although longitudinal studies explicitly exploring mechanisms of influence are lacking, prior research has shown that substance use reduces inhibitions and impairs judgment (Khadr et al., 2016) predisposing to an adverse sexual health outcome, including sexual violence (Martino et al., 2005; Reingle et al., 2012).

### Analytic Techniques

**Fig. 2:**
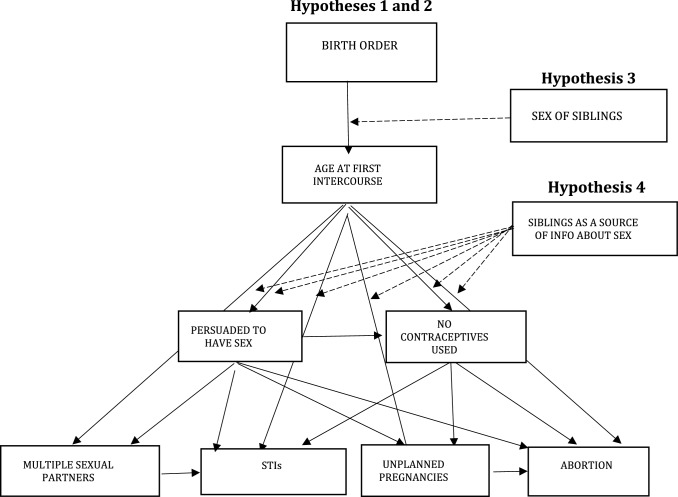
Path diagram. *Note* Black lines represent the main effects (Hypothesis 1 and 2), while dotted lines represent moderator effects (Hypothesis 3 and Hypothesis 4

First, descriptive statistics are provided on study variables for women and men. Then, path analysis is used to simultaneously estimate direct effects of birth order on age at first intercourse and direct and indirect effects of age at first intercourse on both sexual behaviors and sexual health via sexual behaviors (Fig. 2). Analysis of unplanned pregnancies and abortion was restricted to females since we do not have this information for males.

Path analysis was carried out using the STATA 14 gsem command (Huber, 2013), and we controlled for a set of demographic and parental covariates related to the first sexual experience. Structural equation modeling (SEM) is a tool commonly used to elucidate pathways between covariates. Generalized structural equation modeling (GSEM) is an extension of SEM that allows analysis of categorical data. It utilizes a logistic link function that does not rely on linear assumptions to model relationships with dichotomous data. Estimated linear and logit coefficients (Coef.), standard errors (SE) and *p* values (p–v) are reported in the result section and listed in figures. Multiple mediation and moderation analyses were done, respectively, by using the KHB method, available as a STATA command, which allows decomposing the total effect of age at first intercourse on sexual health outcomes into its direct and indirect components (for methodological details, see Karlson et al., 2011; Kohler et al., 2011) and through the use of interaction variables and Wald tests. All estimates were conducted separately by sex using weights that accounted for the survey design and sampling responses so that the sample was representative of the general population of Britain (Erens et al., 2014).

Finally, in order to check the robustness of our findings, we have replicated our analysis using multiply imputed data. We used Multiple Imputation by Chained Equations to impute missing values on outcome and on explanatory variables due to item non-response (Augmented *N* = 1,377 for men and 1,756 for women). We imputed 20 datasets and report consolidated results from all imputations using Rubin's combination rules (see Marchenko, 2009). Results from the imputed analyses did not vary substantively from the analyses using listwise deletion (imputed results are available upon request).

## Results

Table 1 shows descriptive statistics of the analytic sample for men and women. The mean age at first intercourse for both men and women was around 16 years old. Around 38–39% of men and women were the oldest-borns, 34–36% were the youngest, 18% were middle-born and only 7% of the total sample were only children. Women were more than twice as likely to report having been persuaded to have sex at the first time (15% of women compared with 8% of men, *p* < .001) and were four times more likely to report experiencing an STI at 32% compared with 7% of men (*p* < .001). However, a significantly (*p* < .001) higher proportion of men reported having had multiple sexual partners in the last year (43% of men compared with 35% of women). A sibling was a source of information regarding sex for about 15% of participants having at least one sibling, and these were almost entirely from same-sex siblings.

**Table 1:** Descriptive statistics of NATSAL-3 (2010–2012): Mean with SD and the % distribution of variables are reported separately for women and men

	Women		Men		
	*N*	M (SD)	*N*	M (SD)	*p*-value
Age at 1st intercourse	1278	15.93 (1.52)	1073	16.04 (1.66)	
	*N*	%		%	
*Birth order*					
Oldest child	504	39.44	410	38.21	n.s
Youngest child	452	35.37	391	36.44	
Middle child	230	18.00	193	17.99	
Only child	92	7.20	79	7.36	
Persuaded to have sex at the 1st time	190	14.87	90	8.39	***
No reliable contraceptives at the 1st time (condoms. pill. emergency contraceptives or other)	119	9.31	111	10.34	n.s
Multiple partners in the last year	457	35.76	463	43.15	***
Sexually transmitted infections (STIs)	416	32.55	80	7.46	***
Unplanned pregnancy in the last year	41	1.74			
Abortion	123	9.63			
*Sibling(s) as main source of information about sex*					
No	1118	87.48	929	86.58	***
Yes (same-sex sibling(s))	32	2.50	61	5.68	
Yes (opposite-sex sibling(s))	54	4.23	27	2.52	
Yes (mixed-sex siblings)	74	5.79	56	5.22	
*Sex of sibling(s)*					
Only brother(s)–one or more	400	31.30	315	29.36	*
Only sister(s)–one or more	322	25.20	331	30.85	
Mixed-sex siblings–two or more	464	36.31	348	32.43	
Did you smoke cannabis the 1st time you had sex?	15	1.17	22	2.05	*
Were you a bit drunk before having sex the 1st time?	171	13.38	193	17.99	***
Were you not in a relationship with his/her 1st sexual partner?	449	35.13	545	50.79	***
Did you live continuously to age 14 with both parents?	840	65.73	765	71.30	**
Yes					
Large family (3 or more siblings)	58	4.54	46	4.29	n.s
*Household income in £*					
< *2.500*	56	4.38	33	3.08	***
2.500–4.999	74	5.79	37	3.45	
5.000–9.999	125	9.78	55	5.13	
10.000–19.999	173	13.54	129	12.02	
20.000–29.999	125	9.78	112	10.44	
30.000–39.999	117	9.15	104	9.69	
40.000–49.999	84	6.57	82	7.64	
50.000 +	120	9.39	126	11.74	
Not answered	404	31.61	395	36.81	
*Religion*					
None	854	66.82	761	70.92	***
Christian—Church of England/Anglican	387	30.28	33	3.08	
Christian—Roman Catholic	8	0.63	75	6.99	
Christian—other	7	0.55	161	15.00	
Non-Christian	22	1.72	43	4.01	
Ethnicity					***
White/White British	1144	89.51	970	90.40	
Mixed ethnicity	39	3.05	45	4.19	
Asian/Asian British	28	2.19	34	3.17	
Black/Black British	48	3.76	16	1.49	
Chinese /Other	19	1.49	8	0.75	

*Source* NATSAL-3

*Note p* value refers to *χ*^2^ test for categorical variables and to two-sample *t*-test for continuous variables. ***p < .01, **p < .05, *p < .10). *SD* Standard deviation

### Birth Order and Sexual Behavior—Hypothesis 1 and 2

**Fig. 3:**
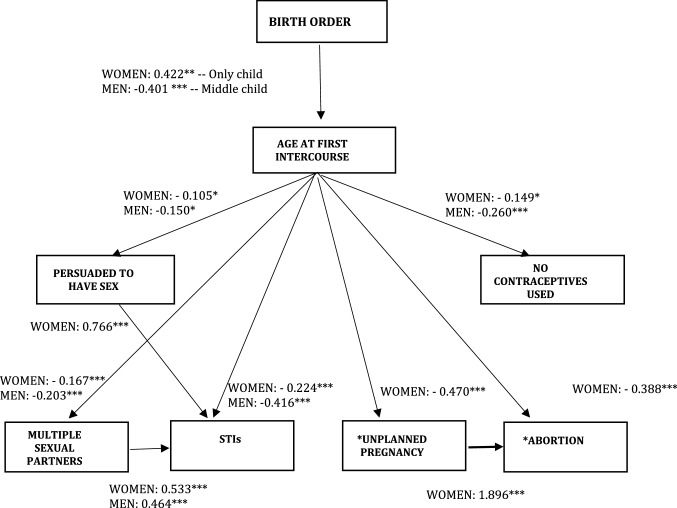
GSEM Results—Hypothesis 1 and Hypothesis 2. *Note* Survey weights are applied. Fully adjusted linear and logit regression coefficients are reported with asterisks as indicators of the significance level: ***p < .01, **p < .05, *p < .10. for women and men. Non-statistically significant paths were dropped out from the figure. Unplanned pregnancies and abortions have been observed only for women. The unweighted sample sizes are 1,278 (women) and 1,073 (men)

Figure 3 shows structural equation estimates for young women and men. Whereas middle-born boys had a significantly younger age at first sexual initiation (Coef. = -0.401; SE = 0.142), only-child women reported higher ages at first intercourse (Coef. = 0.422; SE = 0.196) compared with being the oldest. In turn, older age at first intercourse was significantly associated with a lower likelihood of all the risky behaviors and sexual health outcomes considered for both men and women. Specifically, for both men and women, older age at first intercourse was associated with a significantly lower likelihood of being persuaded to have sex the first time (*p* < .10); not using any form of contraception at their first sexual experience (*p* < .10 for women; *p* < .001 for men); having more than one partner in the last year (*p* < .001); and having been infected by STIs (*p* < .001). In addition, among women, older age at first sexual intercourse was associated with a lower likelihood of having had an abortion, not only directly (*p* < .001) but also indirectly, through a suggestion of a lower likelihood of having had an unplanned pregnancy (KBH test for indirect effect, *p* < .10).

Women who had been persuaded to have sex for the first time were significantly more likely to have STIs (Coef. = 0.766; SE = 0.204) partially mediating the association of age at first intercourse with STIs, although significant at 10% (KBH test, *p* < .10). Having had multiple sexual partners in the last year was also significantly associated with reporting experience of STIs for both men (Coef. = 0.624; SE = 0.291) and women (Coef. = 0.533; SE = 0.154). A formal test for mediation (not reported) showed that 17% of the relationship between age at first intercourse and STIs was explained by multiple sexual partners for women (KBH test,* p* < .01). Similarly, about 11% of the effect of age at first intercourse on STI on men was mediated by multiple sexual partners (KBH test, *p* < .05). Finally, having had an unplanned pregnancy explained almost 5% of the relationship between age at sexual debut and the likelihood of having an abortion (KBH test, *p* < .01).^1^

### Moderation Effect by Siblingship Sex Composition—Hypothesis 3

Table 2 shows findings related to the hypothesized moderation effect due to the siblingship sex composition. An interaction term between siblingship sex composition and birth order was added to the model and was not statistically significant (*p* > .1) for both women and men suggesting the birth order effect on age at first intercourse was not moderated by siblings’ sex composition. Apart from birth order, having mixed-sex siblings was negatively associated with age at first intercourse among male respondents (Coef. = – 0.330; SE = 0.198). Finally, the association between being a middle-born boy and earlier age at first intercourse remained statistically significant independent of the interaction term (Coef. =  – 0.368; SE = 0.322). A similar result was found for only child girls (Coef. = 0.581; SE = 0.215).

**Table 2:** GSEM interaction results—Hypothesis 3

Variables	Women (*n* = 1,278)	Men (*n* = 1,073)
	Age at 1st intercourse	
*Birth order (ref. Oldest child)*		
Youngest child	0.0759	0.079
	(0.158)	(0.186)
Middle child	− 0.146	− 0.638**
	(0.312)	(0.322)
Only child	0.581***	0.172
	(0.215)	(0.259)
*Sex of sibling(s) (ref. Opposite-sex sibling(s))*		
Same-sex sibling(s)	0.214	− 0.183
	(0.175)	(0.185)
Mixed-sex siblings	0.283*	− 0.330*
	(0.162)	
		(0.198)
Interaction term (Birth order * Sex of sibling(s))		
Youngest child* Same-sex sibling(s)	− 0.176	− 0.0324
	(0.248)	(0.277)
Youngest child*Mixed-sex siblings	− 0.306	0.117
	(0.236)	(0.290)
Middle child * Same-sex sibling(s)	0.163	0.664
	(0.440)	(0.429)
Middle child * Mixed-sex siblings	− 0.0963	0.299
	(0.355)	(0.387)

*Source* NATSAL-3

*Note* Survey weights are applied. Fully adjusted linear regression coefficients with standard errors in parenthesis are reported with asterisks as indicators of the significance level: ***p < .01, **p < .05, *p < .10. for women and men

### Moderation Effect of Siblings Information About Sex—Hypothesis 4

Finally, we hypothesized that the association between age at first intercourse, risky sexual behaviors and sexual health would be moderated by having a sibling as the source of information about sex, especially if female.

Concerning women, we found that the magnitude of the negative association between older ages at first intercourse and not using contraceptives at the first time was even greater for those reporting having had sister(s) as their confidante about sex (– 0.119 + (– 1.287) =  – 1.406; *p* < .001). By contrast, having had brothers as a confidant about sex increased women’s probability of having had unprotected sex the first time (1.852; *p* < .001), although at higher ages at sexual debut. Moreover, having had a sister as a referent person in sex education significantly increased the magnitude of the negative association between age at first sex and having had unplanned pregnancies (– 1.453, *p* < .001). Moderations were additionally confirmed by the Wald test (*p* < .001).

Finally, there was also evidence of moderation of the age at first intercourse effect if men had siblings as informants about sex. Specifically, the negative association between age at sexual debut and the use of any kind of contraceptive at the first time was even stronger if they had sisters as a source of information about sex (– 1.068, *p* < .01). At the same time, the negative association between age at first sex and STIs was significantly moderated by having had a sister as a source of information about sex, which significantly decreased the magnitude of this association (– 0.004; *p* < .01). Tables 3 and 4 list findings for women and men, respectively.

**Table 3:** GSEM interaction results in women—Hypothesis 4

Women (*N* = 1,278)						
Variables	Persuaded to have sex	No contraceptive used	Multiple sexual partners	STIs	Abortion	Unplanned pregnancies
Age at 1st intercourse	− 0.120*	− 0.119	− 0.174***	− 0.230***	− 0.491***	− 0.406***
	(0.068)	(0.084)	(0.054)	(0.0514)	(0.093)	(0.183)
*Source of info about sex (ref. no siblings)*						
Yes, opposite-sex sibling(s)	− 0.903	− 33.19***	− 6.248	8.731*	− 4.076	− 4.060
	(11.31)	(7.925)	(5.231)	(5.089)	(4.487)	(4.709)
Yes, Same-sex sibling(s)	1.418	19.20***	5.818	− 6.724*	− 0.00994	16.14**
	(3.928)	(6.674)	(4.445)	(3.859)	(7.025)	(7.833)
Yes, mixed-sex siblings	− 2.392	6.355	− 3.052	0.174	− 2.286	− 14.62
Interaction term	(3.275)	(4.004)	(2.625)	(3.314)	(3.463)	(9.011)
Age at 1st intercourse * opposite-sex sibling(s)	− 0.0274	1.971***	0.406	− 0.567*	0.228	0.302
	(0.725)	(0.490)	(0.335)	(0.332)	(0.277)	(0.304)
Age at 1st intercourse * Same-sex sibling(s)	− 0.0622	− 1.287***	− 0.336	0.438*	− 0.0268	− 1.047**
	(0.243)	(0.448)	(0.282)	(0.237)	(0.455)	(0.531)
Age at 1st intercourse * mixed-sex siblings	0.171	− 0.394	0.193	0.0261	0.158	0.927*
	(0.208)	(0.262)	(0.165)	(0.211)	(0.221)	(0.554)

*Source* NATSAL-3

*Note* Survey weights are applied. Fully adjusted logistic regression coefficients (logits) with standard errors in parenthesis are reported with asterisks as indicators of the significance level: ***p < .01, **p < .05, *p < .10

**Table 4:** GSEM interaction results on men—Hypothesis 4

Men (*N* = 1073)				
Variables	Persuaded to have sex	No contraceptive used	Multiple sexual partners	STIs
Age at first intercourse	− 0.186**	− 0.270***	− 0.195***	− 0.459***
	(0.0832)	(0.0915)	(0.0495)	(0.0985)
*Source of info about sex* *(ref. no siblings)*				
Yes, Opposite-sex sibling(s)	− 3.344	11.47*	− 2.408	− 7.519**
	(5.163)	(6.543)	(3.699)	(3.056)
Yes, Same- sex sibling(s)	− 2.773	− 6.957	− 3.933	− 2.857
	(6.912)	(5.540)	(2.562)	(3.392)
Yes, mixed-sex siblings	− 5.751	− 3.026	4.883	− 6.442*
Interaction term	(5.481)	(3.485)	(3.714)	(3.297)
Age At 1st intercourse * opposite-sex sibling(S)	0.208	− 0.798**	0.159	0.455**
	(0.322)	(0.405)	(0.229)	(0.182)
Age at 1st intercourse * same-sex sibling(s)	0.113	0.415	0.229	0.216
	(0.426)	(0.342)	(0.159)	(0.216)
Age at 1st intercourse * mixed-sex siblings	0.349	0.200	-0.286	0.364*
	(0.340)	(0.220)	(0.238)	(0.210)

*Note* Survey weights are applied. Fully adjusted logistic regression coefficients (logits) are reported with asterisks as indicators of the significance level: *** *p* < .01, ** *p* < .05, * *p* < .10

### Sensitivity Analyses

Sensitivity analyses were conducted by (1) extending the sample to include those who were not yet sexually active by the age of 24 by setting their age at first sex equal to 24 (*N* = 1,729 for men and 2,140 for women), and (2) a survival analysis which handles right-censored data. Results (not shown) were consistent with those reported here for both sexes.

A robustness check was conducted concerning the moderation effect of having siblings as a source of information about sex (Hypothesis 4). Specifically, because the reference group for this analysis is heterogeneous (comprised of a small number of those without siblings alongside those with siblings who were not reported as being a source of information about sex), it could be capturing a sibling effect rather (or in addition to) a “sibling not acting as a source of information” effect. Therefore, we repeated the analysis, confining the sample only to those who had sibling(s) (93%-94% of the analytic sample). Results (not shown) are not significantly different from those done on the entire sample.

## Discussion

This paper investigated the relationship between birth order and timing of sexual initiation, and whether this, in turn, influenced risk-taking behavior and sexual health by elucidating relevant differences between men and women. Overall, although some features of men and women’s sexual debut are rather similar—i.e., age at first intercourse—important differences have been raised about unwillingness of first sexual experience. This information is in line with EU statistics on sexual abuse and violence, showing that 12% of women have indicated having experienced some form of sexual abuse by the age of 15 (FRA-European Union Agency for Fundamental Rights, 2014). Moreover, although rape and the explicit use of pressure to achieve sex (i.e., coercion) is socially and legally unaccepted, coaxing is a more common and generally acceptable behavior in relationships (Camilleri et al., 2009). Policies at both national and international levels should consider empirical evidence about individuals’ experiences of abuse to plan actions fighting violence against women.

Based on Adler’s theory of the relational nature of birth order, with sibling rivalry leading later-borns to be more inclined to adopt risky behaviors (Bank et al., 2004; Sulloway, 1996), we hypothesized that later-borns would be more likely than older siblings to initiate sexual activities at younger ages, with middle-born siblings being the earliest. We evaluated the chain of relationships by looking at the mediation role played by risky behavior related to the first sexual experience and sexual health outcomes. Then, we tested siblings social referencing through the moderation effect played by siblings’ sex composition (“same-sex siblings effect” and “sister effect”) and finally, based on symbolic interactionism emphasizing the relevance of communication among siblings acting as confidants, we hypothesized a potentially moderating effect played by having a sister as the source of information about sex (“mentor effect”).

Our results only provide consistent evidence in support of our first hypothesis for men. Middle-born boys were significantly more likely to initiate sexual intercourse at earlier ages compared with oldest-born siblings. Indeed, with the aim of reducing direct competition, siblings receiving less parental attention may develop different attitudes and qualities that generally make them more rebellious, more likely to be open to new experiences and to adopt risky behaviors (Bu & Sulloway, 2016). Even if in modern societies parents tend to provide equal levels of attention and resources for their offspring, the pattern of parental investment still differs among siblings (Bu & Sulloway, 2016). Thus, because both first- and last-borns experience a period of exclusive parental investment when other siblings are not yet born or have grown up (Bu & Sulloway, 2016), they generally receive a greater share of attention and resources compared with middle-borns.

However, previous research suggests that it is not structural features per se, but mainly the sibling relationship dynamics and the use of them for social referencing (Bandura, 1992) that explain outcomes (Furman & Lanthier, 2002), which likely depend somewhat on the sex composition of the siblingship in the household. Indeed, same-sex siblings in general, and dyads of sisters in particular, may be more likely to share life events and to use each other as a reference person by imitating and modeling behaviors, increasing younger siblings’ early sexual engagement. Thus, as we have specified in our third hypothesis, the siblings’ sex composition may moderate birth order effects on timing of sexual debut, with younger same-sex siblings being more likely to have earlier sexual debut than later-borns of opposite-sex sibling dyads. However, our results did not provide support for this hypothesis (*p* > .10). These findings are particularly interesting since they elucidate the complexity of how family dynamics can affect individual behaviors due to a myriad of combinations of family size, birth order and sex mix.

Regarding the consequences of younger sexual debut, our findings showed that some of the hypothesized associations worked through other sexual risk behaviors, so that early sexual initiation might become a behavioral trigger for an accumulating pathway of risky events. For example, we have shown that part of the association between early sexual initiation and STIs was explained by having had multiple sexual partners for both men and women, and also by having had non-consensual sex at the first time, which explained about 6% of the association for women. These results are consistent with previous studies, showing that early initiators are less likely to obtain, negotiate, or use contraception, to have more lifetime sexual partners, to have an STI, to have unplanned pregnancies and abortions (e.g., Epstein et al., 2014; Sneed, 2009). These findings extend current knowledge concerning the life-course interceding mechanisms between age at sexual initiation and sexual health outcomes.

Although we generally found an absence of sex differences in the effects of early sexual initiation on sexual health outcomes, our estimates show that women who were persuaded or forced to have sex at the first time had a greater risk of contracting STIs compared with men. Prevalence levels of STIs and being forced to have sex the first time also differed for men and women, with women reporting more (*χ*^2^ *p* < .001). Explanations concerning sex differences in reporting STIs mainly arise from physiological factors (Vasilenko et al., 2016) as well as social norms of masculinity prohibiting discussions of sexual health (Knight et al., 2012). Reasons concerning the higher proportion of non-consensual intercourse among women come mainly from a lack of control in the relationship, low self-esteem, an internalized pressure to become sexually active (Finer & Philbin, 2013), and a tendency to engage in sex to please a partner (Vasilenko et al., 2016). Moreover, some evidence has been provided with regard to the role of strict gender roles and stereotypes as relevant determinants of sexual coercion and abuse with some adolescents perceiving violent attitudes and behaviors as an expression of romance and passion (Fonseca et al., 2018).

Finally, we have hypothesized that having a sister as the confidant concerning sexual issues would protect both men and women from adopting risky behaviors and contracting sexual diseases (“mentor effect”). Some evidence concerning a buffering effect of having siblings as main sources of information about sex has been provided. Specifically, our findings elucidated a moderation effect due to having a sister as a reference-person in learning about sexual issues on the link between age of first intercourse and contraceptive use for both women and men and preventing unplanned pregnancies for women. However, the buffering effect did not hold true for STIs among men who had sisters as their source of information regarding sex. Although these finding would need replication in a larger sample to be confident in the effect modification, the results suggest relevant differences with regard to sisters’ and brothers’ power to educate siblings about safe sex practices, stressing the need for a better understanding of siblings’ relationships especially with regard to the sib-ship sex composition. Indeed, our expectations concerning siblings of the same-sex providing greater opportunity for interaction and more parallel socialization histories (Haurin & Mott, 1990; Killoren & Roach, 2014) have been confirmed regarding women. However, findings concerning having a brother acting as a role model need further investigation. Further research is also needed concerning mixed-sex siblings as the moderation effect suggested the existence of an unobserved “large-family” effect since the number of siblings could significantly influence the quality of family interaction and communication (Elton et al., 2019). An explanation for why having brothers as a reference person for a woman would lead to a higher risk of engaging in risky sexual behaviors arises from the tendency toward a larger number of sexual partners. According to the theory of opportunity, mixed-sex siblings with high level of confidence and closeness are likely to share life events as well as the same friends, which may bring more sexual opportunities due to a higher exposure to potential opposite-sex partners.

However, our findings seem to reflect the idea that, in general, girls may be effective teachers to their younger siblings (Widmer, 1997), not only on using contraceptives or avoiding unplanned pregnancies but also in reporting STIs. Indeed, the lower rate of STI reported among young men and, in general, their disengagement from sexual health services, has been linked to enactments of masculinity that prohibit discussions of sexual health (Knight et al., 2012). Some authors relate men’s reticence to engage in discussions about sexual health to dominant masculine ideals that prescribe stoicism, independence, self-reliance and a lack of interest in self-health. Thus, our finding suggests that having a sister as informant about sex might have a positive effect in dealing with STIs among early initiators because these young men may be less “contaminated” by the masculinity culture that makes them less prone to reporting their sexual health problems. Despite decades of public health intervention, STIs remain a serious health problem, especially among young men due to their resistance to declare them.

Some limitations of our study must be acknowledged. Information on the quality or specific content of sibling interactions as well as on sibling risky behavior itself was not available in this data set. We did not have any information about spacing between siblings, and although we deal with sex composition of siblingships, we cannot precisely identify whether the sibling providing information about sex was an older sibling or not. In addition, information concerning family norms and beliefs about sex was not available as well as information regarding parenting styles and social support. Moreover, since the analyses might be not be free of endogeneity, the results only refer to associations between variables and, despite the practice of reporting “effects” in path models, they should not be interpreted in terms of causation. Although the sample was age-restricted, there may still be some issues concerning recall bias regarding the age of sexual initiation, in that those with adverse sexual experiences may recall them differently from those without**.** Indeed, self-reported information about sensitive aspects of life might be underreported or forgotten. However, this potential under-reporting means that estimated effects are potentially downwardly biased so any arising conclusions can be interpreted as reliable. Finally, the paper only refers to heterosexual vaginal intercourse and omits information about homosexual experience, for which we had too few observations, and other sexual debut experiences such as oral and anal sex. Indeed, the exclusion of people with diverse genders and sexualities (e.g., transgender, non-binary) represents an important limitation of this study. Future work should specifically address these dynamics in more diverse samples. Although participants with missing data were omitted from the analyses, perhaps introducing bias into the sample, the robustness of results has been checked by replicating GSEM estimates on a complete sample with a multiply imputed analysis. Moreover, a sensitivity analysis did not find evidence of bias from restricting the sample to those who were sexually active by the age of 24 years.

### Conclusion

Sexual activity is a fundamental component of human health and well-being and the foundation of most adult partnerships as well as a prerequisite for natural conception (Johnson et al., 2005). Scholars and policymakers in Europe have paid increasing attention to the relationship between individual sexual behavior and health outcomes, implementing interventions and policies at both the individual and population levels, but a range of social and demographic factors continue to influence sexual behavior (Epstein et al., 2014). Our findings have implications for preventive programs aimed at promoting healthy sexual debut and behaviors over the life span rather than promoting abstinence messages. Indeed, while there is little evidence of the efficacy of abstinence-only education policies in delaying initiation of sexual intercourse, more comprehensive reproductive programs have successfully delayed initiation of sexual intercourse (Santelli et al., 2006). Thus, expanded understanding of the buffering role of communication about sex (the “mentoring effect”) should help practitioners and educators in targeting interventions more successfully.

The results provided here suggest that it is important to investigate further the role of siblings as confidants about sexuality, elucidating underestimated aspects of intimate communication between both same- and opposite-sex siblings. These findings point to the importance of including family members, particularly siblings, in sexual health promotion, education and services rather than trying to delay the age at sexual debut (Kantor et al., 2008). Specifically, siblings could be given a leading role in family-oriented prevention programs aimed at reducing adolescent pregnancy, abortion and STIs or they could be supported and encouraged to be mentors and confidants for each other.

This study has several important strengths. It extends previous work which has almost exclusively compared siblings’ behaviors (e.g., Haurin & Mott, 1990; McHale et al., 2009; Widmer, 1997) focusing only on direct mechanisms of sibling influence through social learning. This is the first study to investigate the effect of birth order on age at the first sexual experience that adopts a perspective that views birth order as a result of specific family dynamics within a comprehensive analytic framework, simultaneously investigating causes and consequences of ages at first intercourse. In addition, most prior work has been set in the U.S. (e.g., Haurin & Mott, 1990; McHale et al., 2009; Widmer, 1997). To our knowledge, this is the first UK study looking at the relationship between birth order, risk-taking behavior and sexual health and has the advantage of using the Natsal-3, a national study which provides detailed information for a large and representative sample. Moreover, the paper has provided evidence concerning the role of siblings, especially if girls**,** as key agents in setting standards of conduct and in protecting one another from risky sexual behavior.

Future studies should better identify contents of communication about sex among siblings and to whether this complements or competes with parents’ involvement in adolescents’ sexual education. In addition, since recent studies have found that adolescents’ communication about sex with siblings has a more limited contribution than peers (Friedman et al., 2019), future studies could elucidate specific patterns of influence in only-child adolescents for whom peers might substitute for siblings’ mentoring. Overall, more evidence is needed on how the influence is effected and on the level of intimacy needed in interpersonal relationships. Moreover, future studies need enough information to include people with diverse gender and sexuality. Finally, longitudinal studies are needed to explore better the social and psychological pathways explaining risky sexual experiences, for example by characterizing substance use as a mechanism that reduces inhibitions, given the evidence of the link with adverse sexual health outcomes (Martino et al., 2005; Reingle et al., 2012). Siblings are a fixture in the family lives of children and adolescents being both companions and confidants (McHale et al., 2012), and while they sometimes act as combatants and antagonists, they remain an enduring and important source of social learning. Our findings suggest that the timing of events is very important and thus, when and how becoming sexually active can have long-term consequenes for sexual health later in life.

## Footnotes

1 Since in our sample there are any observations reporting to have had an abortion and to belong to Roman Catholic religion, we excluded the religion variable as control when we estimated KHB method.
